# Advances in the management of intraocular foreign bodies

**DOI:** 10.3389/fopht.2024.1422466

**Published:** 2024-09-02

**Authors:** Marc Ohlhausen, Bryant A. Menke, Jack Begley, Sean Kim, Matthew R. Debiec, Christopher D. Conrady, Steven Yeh, Grant A. Justin

**Affiliations:** ^1^ Department of Ophthalmology, Truhlsen Eye Institute, University of Nebraska Medical Center, Omaha, NE, United States; ^2^ Department of Ophthalmology, Walter Reed National Military Medical Center, Bethesda, MD, United States; ^3^ Department of Pathology, Microbiology, and Immunology, University of Nebraska Medical Center, Omaha, NE, United States; ^4^ National Strategic Research Institute, University of Nebraska Medical Center, Omaha, NE, United States; ^5^ Global Center for Health Security, University of Nebraska Medical Center, Omaha, NE, United States

**Keywords:** intraocular foreign body, ocular trauma, vitrectomy, open globe injury, endophthalmitis

## Abstract

Intraocular foreign bodies (IOFBs) remain a severe complication of ocular trauma commonly encountered worldwide. This literature review aimed to discuss current practice patterns, areas of controversy, and advances in the management of IOFBs. Injuries involving IOFBs carry significant ocular morbidity and management can be extremely challenging. A systematic approach to preoperative evaluation and IOFB surgical management is detailed in this article and should be applied in each case. The location and composition of an IOFB have important implications on surgical approach and timing, especially in cases of toxic metals and vegetable matter. The advantages, disadvantages, and previous literature regarding immediate versus delayed foreign body removal are presented. Surgical approaches are described, with an emphasis on posterior chamber IOFB management and removal via pars plana vitrectomy. Final visual acuity is variable, but approaches have been used to prognosticate outcomes including the Ocular Trauma Score. By synthesizing current IOFB literature, the goal is to provide practitioners with guidance that will maximize the chances of surgical success and patient outcomes.

## Background

Traumatic eye injuries remain a significant cause of vision loss within the United States and worldwide. Recent studies have estimated that the incidence of open globe injuries (OGIs) in the United States is around 4.5 per 100,000 population each year (1). It is estimated that intraocular foreign bodies (IOFBs) are found in 18-41% of OGIs (2, 3). According to data from the 2019 Global Burden of Disease Study, the global age-standardized incidence rate (ASIR) of intraocular foreign bodies decreased <1% from 1990 to 2019. However, from 2008-2019 global incidence trended upwards from around 350 cases per 100,000 in 2008 to over 450 cases per 100,000 in 2019 (2). The highest incidence rates are found in developing countries (2). Developing countries often have a higher proportion of workers in manufacturing and agriculture, which are two of the job sectors in which workers are most prone to suffering traumatic eye injuries (2, 4, 5).

Various demographic and environmental characteristics have been associated with an increased incidence of IOFBs. According to the US Eye Injury Registry, IOFBs occur most commonly in men between the ages of 21 and 40 years old. These incidents often occur at work (54-72%) and at home (30%), with the most common mechanisms involving hammers, power tools, and weapons/explosives-related injuries (3). A predominance of young males working manual labor jobs was also seen when examining IOFB patients in Ireland, Greece, and China (5–7). Often, these patients are not wearing eye protection at the time of their trauma (5, 6, 8).

IOFBs may be classified based on location and substance, which may be unknown or presumed at the time of the initial injury. Distinguishing whether the IOFB is in the anterior or posterior segment, as well as whether the lens is involved, is important for surgical planning. The majority of IOFBs reside in the posterior segment of the eye (58%-88%), followed by the anterior segment (10%-15%) and the lens or orbit (2%-8%) (3, 9). The most frequent substances that constitute IOFBs are metals such as iron, copper, lead, zinc, aluminum, and nickel. Certain metals can lead to conditions such as siderosis bulbi (iron) and chalcosis (copper). Inert substances like glass, wood, concrete, and plastic are also frequently involved. Organic substances such as vegetable matter, insects, and animal hair can be particularly troublesome, as they confer an increased risk of endophthalmitis compared to other IOFBs (10–12).

Most sources estimate the overall risk of endophthalmitis for all retained IOFBs to be between 5% and 30% (10, 13). The risk of endophthalmitis occurrence was found to be about 6.5% when averaged across IOFB literature from the past 30 years as reviewed by Colyer et al. (10, 11, 14). This compares to acute postoperative endophthalmitis rates of around 0.1% following cataract surgery and 0.05% following pars plana vitrectomy (PPV) (15–17). In 2008, a retrospective study by Chaudhry et al. showed that a delay of over 48 hours in repairing an OGI and removing an IOFB was associated with an increased risk of developing endophthalmitis and poor visual outcomes (18). Development of endophthalmitis in these cases can be especially worrisome, as post-traumatic endophthalmitis is often associated with a more severe course compared to other etiologies (19, 20). Injuries in rural settings have been associated with higher rates of endophthalmitis, including *B. cereus* endophthalmitis (21, 22). Disruption of the crystalline lens and delayed primary wound closure have also been associated with higher rates of endophthalmitis (22). Early primary repair (within 24 hours), intraocular tissue prolapse, and self-sealing wounds were found to be independent protective factors against the development of endophthalmitis following OGIs (23).

**Figure 1:**
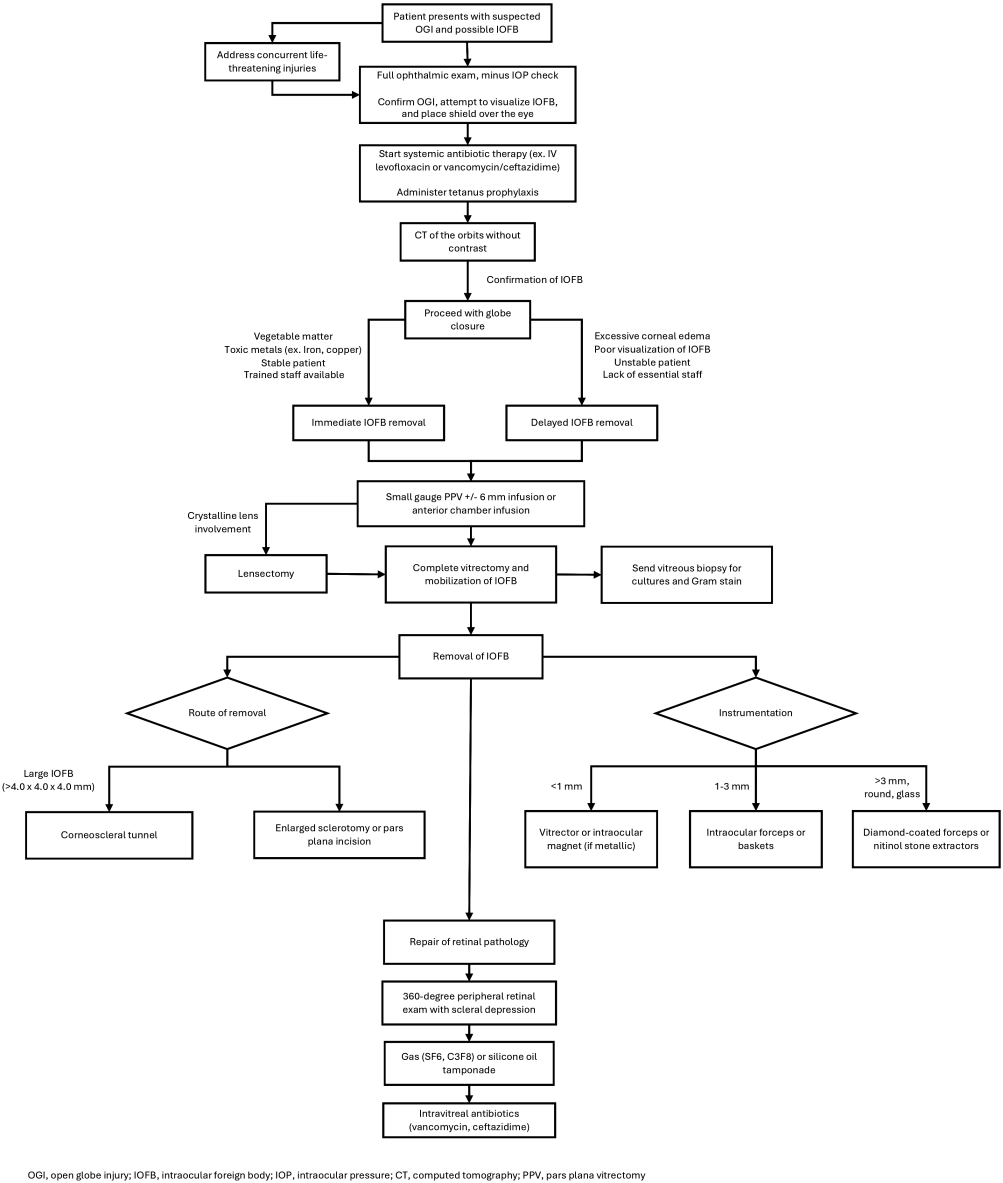
Intraocular foreign body (IOFB) management and decision-making algorithm.

The location and composition of the IOFB have implications on the need for removal. Organic substances and toxic metals generally require immediate extraction while other substances can potentially remain in the eye for longer periods (10, 11). The decision regarding optimal time frame for removal should involve careful consideration of factors such as size, location, and composition of the foreign body, mechanism of injury, and individual capabilities of the surgical team (21). Overall, the primary goal in initial globe injuries with a known or suspected IOFB injuries is to achieve globe closure for IOFB extraction (**Figure 1**). Removal of the IOFB and repair of additional intraocular pathology are secondary but key objectives that may need to be addressed in the near future if unable to be safely accomplished during the initial surgery, either due to poor visualization of the IOFB (i.e., corneal edema or media opacity) or general health status of the patient in polytrauma settings.

## Management and preoperative planning

Open globe injuries can be classified in terms of Zone of Injury (ZOI) based on the location of the rupture. Zone 1 includes the cornea and limbus, Zone 2 includes the 5 mm posterior to the limbus, and Zone 3 contains the remaining globe posterior to 5 mm from the limbus. Zone 3 can be further broken down into 3a and 3b, which is 5 to 8 mm posterior to the limbus and greater than 8 mm posterior to the limbus, respectively. Zone 3 injuries and more severe injuries (larger wound size and presence of retinal detachment) have been associated with the worst visual prognosis and requiring the highest rates of enucleation (23–29). The Ocular Trauma Score (OTS) was proposed by Kuhn et al. as a tool to predict final visual acuity following ocular injury (30). Variables included in the scoring system include initial visual acuity, globe rupture, endophthalmitis, perforating injury, retinal detachment, and a relative afferent pupillary defect (RAPD). Presence of any of these variables, or worse baseline visual acuity, portends an unfavorable final visual acuity outcome. Preoperatively, establishing Zone of Injury and OTS can be useful for prognostication of future visual acuity and counseling patients on likely outcomes. In this way, the surgeon can manage patient expectations early.

Initial evaluation for suspected IOFB includes measuring visual acuity, assessment for the presence of afferent pupillary defects, and a careful pupillary exam, noting the size and shape. Eyelid and eyebrow examination should be used to look for external foreign bodies. Careful examination using slit lamp biomicroscopy is essential. The identification of scleral or corneal lacerations, a positive Seidel test, and uveal prolapse confirm the diagnosis of OGI and may suggest a possible IOFB. Iris transillumination defects can also be a sign of occult globe rupture and IOFB. Identification of an entry site may help to localize the intraocular location of the IOFB.

A detailed examination of the posterior segment is critical, as most foreign bodies are identified here (3). However, occult IOFB injuries may also occur and present as delayed onset intraocular inflammation, sometimes with a granulomatous appearance (21, 31, 32). As with any possible OGI, the examiner should generally avoid checking intraocular pressure, or any examination technique that puts pressure on the globe, until the wound has been closed to prevent further damage to intraocular structures or the extrusion of ocular contents. In cases of iris prolapse, there are some who advocate for the deferral of pharmacologic mydriasis to avoid re-opening the defect. It is also important, especially when the IOFB is related to a blast explosion or military shrapnel, to thoroughly examine the fellow eye for occult injury and treat concurrent life-threatening injuries (14, 33–35).

Detection and localization of the IOFB is critical for surgical planning and treatment. In some cases, whether due to lens opacification, corneal damage, hyphema, or vitreous hemorrhage, the IOFB may be difficult to visualize. In up to 55% of patients, clinical eye exam may not detect the presence of IOFB (36). In these cases, an X-ray of the orbits has been used to localize metallic foreign bodies, but visualization of radio-lucent material is limited (37). For this reason, X-ray of the orbits has been replaced by computed tomography (CT) as the imaging modality of choice in cases of suspected IOFB. CT of the orbits without contrast can identify IOFB in up to 95% of patients (36, 38). One limitation of CT is that manual measurements of IOFB dimensions from the scans has been shown to be inconsistent and often inaccurate (39).

Gentle B-scan ultrasonography can also identify foreign bodies in the posterior segment in up to 52% of patients with IOFB but must be performed with extreme caution (36). Ultrasonography may be especially useful intraoperatively to localize IOFBs that have settled anteriorly in the pars plana and are difficult to directly visualize. Limitations of B-scans include their operator-dependent nature as well as potential for expulsion of intraocular contents if excessive pressure is placed on the globe from the probe (40). Magnetic resonance imaging (MRI) can be useful in detecting organic material, but is contraindicated when metallic foreign bodies are suspected, as the IOFB can dislodge and cause additional ocular damage (41).

Initial management of IOFBs include a shield over the affected eye, intramuscular tetanus toxoid prophylaxis (0.5 mL), antiemetics as needed for nausea control, adequate patient analgesia, and consideration of broad-spectrum intravenous (IV) antibiotics for prevention of endophthalmitis. Typically, patients will be started on initial IV antibiotic therapy immediately following diagnosis, then subsequently transition to oral medication at discharge to complete their antibiotic course. Total course length varies, but typically spans from 48 hours to 7-10 days (42).

Fluoroquinolones such as levofloxacin and moxifloxacin have been found to achieve sufficient aqueous and vitreous humor concentrations to inhibit the growth of 90% of major ocular pathogen isolates (MIC90) when administered in IV or oral forms (43, 44). As such, they are a popular option for antibiotic prophylaxis (45). Typical dosage (both IV and oral) for levofloxacin and moxifloxacin is 500 mg daily and 400 mg daily, respectively, and is usually continued for 1 week of total coverage. An advantage of fluoroquinolones is that they can be used in penicillin-allergic patients but should be avoided in pediatric patients due to concern for arthropathy (46). Another commonly utilized antibiotic regimen is IV vancomycin (1 gram every 12 hours) for gram-positive coverage, combined with a cephalosporin for gram negative coverage such as the third-generation cephalosporin ceftazidime (1 gram every 8 hours) (22, 45, 47). Cefazolin and cefepime have also been used in some studies (48, 49).

Definitive IOFB management involves globe closure and IOFB removal, although the timing of foreign body removal is up for debate (50, 51). Immediate removal, classified as removal of the IOFB during the same surgery as globe closure, possibly has shown a decreased risk of endophthalmitis, proliferative vitreoretinopathy, and post-traumatic endophthalmitis compared to delayed removal; although it has not been associated with significant visual improvement (21, 52). In a recent study, the incidence of postoperative endophthalmitis following surgical repair of globe injury combined with IOFB removal within 24 hours of initial injury was found to be 3.70% (53).

Importantly, in a study of 79 eyes during Operation Iraqi Freedom and Operation Enduring Freedom, military personnel had delayed removal of IOFBs on average of 21 days following initial injury and there were no cases of endophthalmitis (14). Almost all (97%) of these patients received systemic (86%) and/or topical (85%) antibiotic coverage during this time. These findings demonstrate that there may be minimal risk of endophthalmitis with delayed IOFB removal and adequate antibiotic coverage in combat ocular trauma (14, 54). Of note, all IOFBs in this study were a consequence of exploding ordnance. The subsequent high-energy projectiles from these explosions may become sterilized by the high heat, resulting in the decreased risk of endophthalmitis. Delayed foreign body removal allows time for intraocular inflammation and corneal edema to improve, resulting in superior visualization during surgery, along with providing time for the possible formation of a posterior vitreous detachment (PVD) (14, 50).

The ultimate decision of when to remove an IOFB will depend on the patient’s physiologic stability, composition of IOFB, nature of injury, and availability of trained personnel. When signs of endophthalmitis are present, immediate surgical removal of the IOFB is indicated unless a simultaneous life-threatening injury is a contraindication for surgery (3). In cases of hemodynamic instability, delaying removal is appropriate. As mentioned previously, toxic metals, such as iron and copper, and organic FBs are always indications for immediate removal, whereas inert materials such as plastic and glass can remain in the eye for longer periods with fewer complications (4, 55).

## Surgical management

Surgically, a variety of strategies have been employed to successfully remove IOFBs. A specially tailored approach is required for each situation based on the location, size, and composition of the foreign body. Globe closure should be accomplished prior to attempted removal of IOFBs. For both anterior and posterior foreign bodies, the entry site should be identified and prolapsed tissue excised or reposited into the eye prior to closure. Corneal lacerations are most often repaired using 10-0 nylon suture, while 9-0 nylon suture is used to reapproximate the limbus, and 8-0 nylon suture is typically used for scleral wounds. If the IOFB is embedded in the anterior segment, removal is often relatively straightforward. Intraocular forceps or magnets may be used to extract the IOFB through a secondary corneal limbal incision (56). Lensectomy may also need to be completed if the crystalline lens is involved, with placement of an intraocular lens to be considered at a later date (57).

For posterior chamber IOFBs, the current mainstay intervention is PPV with simultaneous removal of the IOFB. PPV also allows the surgeon to address any retinal defects or vitreous hemorrhage that may be present, along with lensectomy if needed. Three port, small gauge (23, 25, or 27 gauge) vitrectomy is the standard approach. An infusion cannula with a 6-millimeter tip is helpful in cases of poor visualization of the posterior segment or choroidal hemorrhage (21). Anterior chamber infusion is another option in these situations. Core vitrectomy is performed, followed by removal of cortical vitreous and induction of posterior vitreous detachment, if necessary. A vitreous biopsy may be taken for Gram stain and culture. The vitreous and potentially fibrous attachments of the IOFB can then be cut circumferentially, allowing for mobilization. If the IOFB is embedded in the retina or choroid, it may be necessary to significantly increase intraocular pressure for a short period to tamponade hemorrhage upon dislodgement of the foreign body.

**Figure 2:**
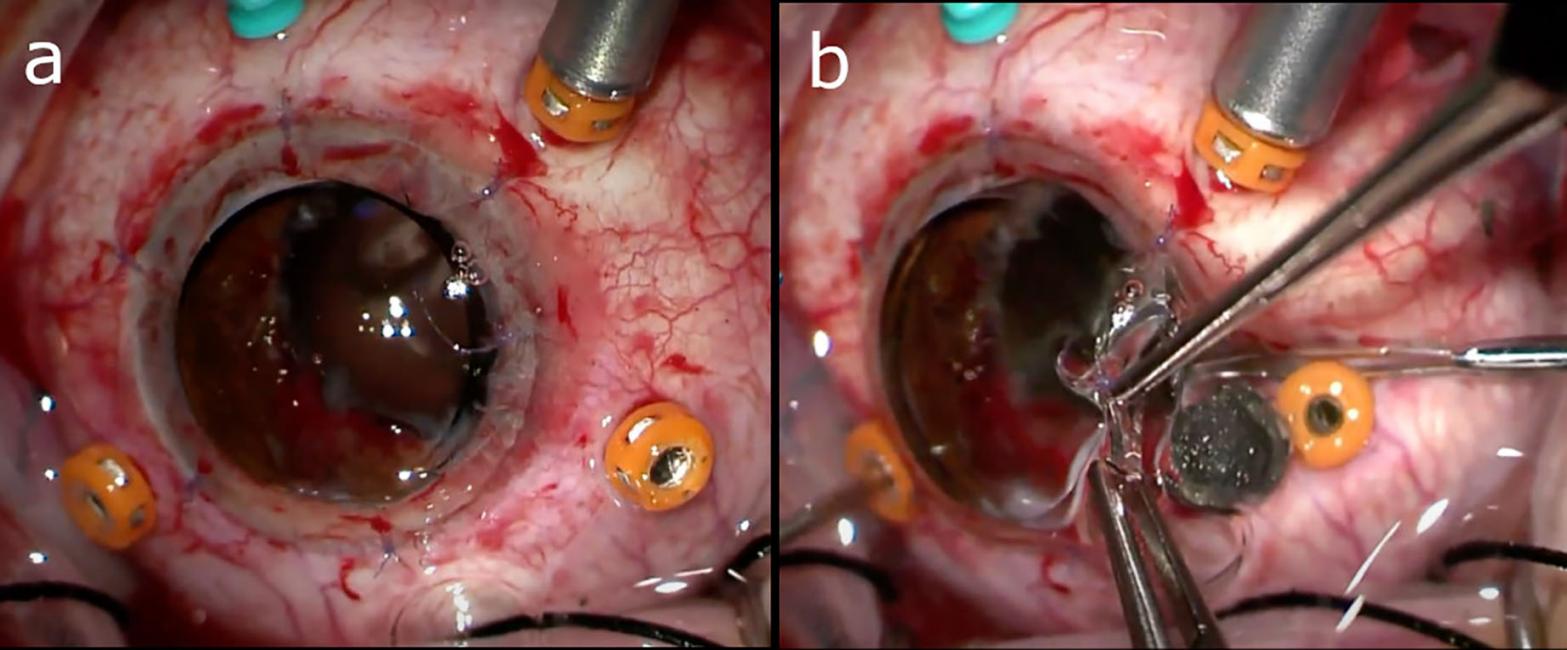
**(A)** Utilization of a temporary keratoprosthesis (TKP) during pars plana vitrectomy (PPV). **(B)** Intraocular foreign body (IOFB) being removed from under the edge of the TKP. Images courtesy of Drs. Grant Justin and Xi Chen.

In cases of significant corneal opacification such that there is insufficient visualization of the posterior segment to undergo PPV, a few different strategies may be employed to accomplish the vitrectomy and foreign body removal. One approach involves working in conjunction with a cornea specialist to remove the opacified cornea and utilize a temporary keratoprosthesis (TKP) during the PPV (**Figure 2**), with completion of the penetrating keratoplasty (PKP) at the conclusion of the case. Although, there has been an association between combined vitreoretinal surgery with TKP and subsequent PKP failure, especially if silicone oil is employed (14, 58, 59). So, if possible, it may be favorable to delay surgery until the native cornea clears rather than proceeding immediately to keratoplasty. Another option is completing the PPV via an endoscopic approach, which is especially useful for visualizing the anterior vitreous and pars plana. And lastly, some have described successfully using B-scan ultrasound-guided vitrectomy in cases where patients are not candidates for a TKP and endoscopy is not available (60).

An alternative strategy for posterior segment IOFB removal more commonly used prior to the advent of modern vitrectomy techniques is an external approach using electromagnets. In these cases, the foreign body is initially localized and brought to an extraction site using the electromagnet on the outside of the eye. The overlying sclera is then incised, and if necessary, prolapsed vitreous is cut and choroidal tissue is cauterized. This approach is no longer commonly utilized and is not recommended especially for posterior segment foreign bodies, as it has been associated with significant iatrogenic injury. When compared to PPV, electromagnet removal led to significantly worse functional and anatomical outcomes (61). There were higher rates of endophthalmitis, vitreous hemorrhage, and proliferative vitreoretinopathy (PVR) in IOFB eyes treated with the electromagnet versus PPV (61).

If an IOFB is large, typically greater than 4.0 x 4.0 x 4.0 mm, then the best avenue for removal is through a corneoscleral tunnel (21, 62). A large pars plana incision can increase the risk of retinal incarceration, vitreous hemorrhage, and retinal detachment (58). For smaller IOFBs, removal can be accomplished through enlarged sclerotomy sites or pars plana incisions (63). The presence of a crystalline lens would preclude removal of the IOFB through the anterior chamber and favor extraction through an enlarged scleral opening as well (50).

To protect the macula from dropped IOFBs, perfluorocarbon liquid (PFCL) can be injected intraoperatively to act as a shield (50, 64). Facilitated by its characteristics of high specific gravity and surface tension, PFCL has been successfully shown to redirect dropped foreign bodies towards the peripheral retina (65). Although, this technique is controversial and there has been evidence from some models that if the foreign body manages to enter the PFCL it may accelerate its descent towards the macula (66). Care must be taken to avoid subretinal migration of PFCL in cases with retinal tears or detachments. Another method of macular protection involves preserving a small area of coagulated vitreous hemorrhage overlying the macula to shield against falling IOFBs. This strategy may only be an option in cases of delayed IOFB removal, as the hemorrhage needs time to coagulate.

Once the IOFB has been removed, a 360-degree peripheral retinal exam with scleral depression should be completed. All retinal breaks should be thoroughly treated with laser or cryotherapy, and gas or oil tamponade may be used at the conclusion of the case. The routine use of prophylactic scleral buckles is a subject of debate. There is some evidence that it may help prevent retinal detachment in cases of IOFBs removed via PPV (67). Prophylactic scleral buckle use in posterior segment open globe injuries has also been associated with improved final visual and anatomical outcomes, along with a non-significant decrease in subsequent retinal detachments (68).

Intravitreal antibiotics are typically instilled at the conclusion of the case for endophthalmitis prophylaxis. A commonly used formulation is intravitreal vancomycin (1.0 mg/0.1 mL) along with ceftazidime (2.25 mg/0.1 mL) to cover for both gram-positive and -negative bacteria (3, 21). In addition, in areas where fungal infections are common, intravitreal voriconazole (50-100ug/0.1mL) or amphotericin B (5-10 ug/0.1mL). Antimicrobial coverage can be adjusted as needed based on Gram stain and culture results.

## Instrumentation

For small IOFBs (<1.0 mm), removal may be accomplished using solely the vitrector or intraocular magnet (if metallic) (21). Intermediate-sized (1.0-3.0 mm) IOFBs may be more amenable to removal with intraocular forceps or baskets. It is often difficult to remove an IOFB using solely an intraocular magnet, as the IOFB may become dislodged during passage through the sclera or cornea and drop to the back of the eye causing iatrogenic trauma to the retina. Instead, magnets may be used to pass the IOFB off to intraocular forceps or baskets which have a more secure grasp. Diamond-coated forceps improve gripping potential, as the diamond splinters are able to dig into the surface of any material and facilitate removal of large IOFBs and those with smooth surfaces such as glass (69).

An emerging tool used for particularly challenging IOFBs is the NCircle^®^ nitinol tipless stone extractor (70). This instrument was originally produced for capture and removal of renal calculi in the ureter and kidneys. Nitinol is a nickel-titanium alloy that is extremely flexible. It is classified as a shape memory alloy, meaning that it returns to its original shape when deformed and is resistant to kinking (71, 72). Four nitinol wires make up the basket of this device, which can be advanced or retracted with a thumbwheel. The basket is inserted into the eye with the wires retracted into the sheath, but as the foreign body is approached the sheath can be retracted and the basket will open. The IOFB is then maneuvered within the basket, the sheath is advanced, and the wires are tightened around the IOFB. This mechanism allows for a secure hold that is unlikely to dislodge while exiting the eye. The nitinol basket is especially useful for IOFBs that are difficult to grasp due to an irregular or round shape, large size, or smooth surface, such as glass (71, 73). Forceps, and magnets for ferromagnetic IOFBs, are helpful for maneuvering an IOFB into the nitinol basket. The long reach of this device, as it was meant to extend deep into the urinary system, means that there will be no difficulty reaching the posterior pole of eyes with even the most extreme axial lengths.

## Published outcomes

At least 21% of eyes with IOFBs have a final visual acuity worse than 20/200 (9, 21, 25, 74, 75). As discussed previously, the Ocular Trauma Score can be used to reliably predict the final vision of the injured eye (25, 76, 77). Many additional factors have been associated with poor visual prognosis, including age greater than 50, worse initial visual acuity, hyphema, vitreous hemorrhage, uveal prolapse, retinal detachment, afferent pupillary defect, vitreous hemorrhage, retinal hemorrhage, complications of retinal breaks, and intraocular perfluoropropane (C_3_F_8_) gas tamponade (3, 9, 21, 56, 78–80). Additionally, central corneal perforations, corneoscleral lacerations, larger IOFB, and IOFBs in the posterior segment are associated with worse visual outcomes (3, 24, 29, 75, 81–84). Higher rates of postoperative proliferative vitreoretinopathy (PVR) and phthisis bulbi have also been associated with IOFB (33). Factors associated with improved visual outcome include better presenting visual acuity, absence of retinal breaks, absence of vitreous hemorrhage, wound length less than 4 mm, and non-vitrectomy surgery (9).

Importantly, patients with a history of IOFBs should be counseled on proper protection of the eyes when working with chemicals, lasers, metal, UV equipment, and other high-risk exposures. In patients with poor visual outcomes, extensive time should be spent with the patient discussing appropriate monocular precautions with 3 mm polycarbonate lenses and safety frames that should be worn at all times. A referral to a low vision specialist can help patients maximize their remaining vision and adapt to their new visual baseline.

Additionally, patients should be evaluated for mental illness related to the trauma. Traumatic open globe injuries have been associated with a high prevalence of anxiety of depression in both adults and children (85, 86). In a small cohort of children, 15% of patients developed generalized anxiety disorder, post-traumatic stress disorder, and depression following an episode of ocular trauma (86). By keeping this potential sequela in mind, practitioners will be more likely to identify patients who would benefit from referral to mental health specialists during post-operative visits. Evaluation and treatment of any mental health disorders associated with the traumatic experience of an IOFB is critical.

## Conclusions

In this review, we provide a synthesis of the literature on the characterization, incidence, preoperative management, and surgical approach of IOFBs. Over half of IOFBs occur in the occupational setting, so encouraging eye protection when at risk is paramount. When there is concern for IOFB, it is critical that a thorough, yet delicate, eye examination be completed using slit lamp biomicroscopy and indirect ophthalmoscopy to identify the IOFB and any associated injury. To assist with surgical planning and location of the IOFB, the first-line imaging in these patients is CT of the orbits without contrast. The surgical removal of an IOFB can be done in an immediate or delayed fashion after globe closure is accomplished, taking into account overall patient health, visibility of the IOFB for immediate extraction, and the environment of care. The surgical approach and timing for IOFB removal is highly variable based on clinical circumstances, with PPV being the mainstay for posterior segment IOFBs. One emerging device for improved removal of hard to grasp IOFBs is the NCircle ^®^ nitinol stone basket. Further investigation is needed to better understand the acute and chronic complications of IOFBs, the factors leading to improved visual outcomes, and ideal surgical approaches in these patients. A better understanding of these will improve our ability to identify and treat patients in the event of ocular injury due to IOFBs.

## Author disclaimer

The view(s) expressed herein are those of the author(s) and do not reflect the official policy or position of the U.S. Army Medical Department, U.S. Army Office of the Surgeon General, Department of the Army, Department of Defense, or any other agency of the U.S. Government. The content is solely the responsibility of the authors and does not necessarily represent the official views of the National Institutes of Health or the views or policies of the Department of Health and Human Services, nor does mention of trade names, commercial products, or organizations imply endorsement by the U.S. Government.
